# A Long-Term and Large-Scale Real-World Study in Taiwan: Efficacy of Target Therapy in Stage IV Colorectal Cancer

**DOI:** 10.3389/fonc.2022.808808

**Published:** 2022-03-17

**Authors:** Sheng-Chieh Huang, Chun-Chi Lin, Hao-Wei Teng, Hung-Hsin Lin, Shih-Ching Chang, Yuan-Tzu Lan, Huann-Sheng Wang, Shung-Haur Yang, Wei-Shone Chen, Jeng-Kai Jiang

**Affiliations:** ^1^ Division of Colon & Rectal Surgery, Department of Surgery, Taipei Veterans General Hospital, Taipei, Taiwan; ^2^ School of Medicine, National Yang-Ming University, Taipei, Taiwan; ^3^ Division of Medical Oncology, Department of Oncology, Taipei Veterans General Hospital, Taipei, Taiwan

**Keywords:** colorectal cancer, metastatic colorectal cancer, target therapy, cetuximab, bevacizumab, Taiwan, real-world study, metastectomy

## Abstract

This study expands the understanding of the role of target therapy in improving survival of patients with mCRC based on real-world study results. These data represent potential survival outcomes of Taiwanese patients with mCRC in clinical practice. CRC is the most commonly diagnosed cancer and the third leading cause of cancer-related death in Taiwan. The aim of this study was to evaluate the efficacy of target therapy in combination with chemotherapy for mCRC in Taiwan. This was a real-world, retrospective, observational study in patients diagnosed with mCRC (N=1583). A total of 792 patients received chemotherapy plus target therapy (anti-EGFR therapy, n=180; anti-VEGF therapy, n=612) and 791 patients who received chemotherapy alone. Overall survival (OS) and progression-free survival (PFS) were examined. For RAS wild-type patients, the median OS (mOS) was 34.3 months in the EGFR L (left-sided colon) group, 27.3 months in the VEGF L group, 18.4 months in VEGF R (right-sided colon) group, and 13.8 months in EGFR R group (*P*<0.001). Median PFS (mPFS) was 9.8 months in the EGFR L group, 8.9 months in the VEGF L group, 6.8 months in VEGF R group, and 5.8 months in EGFR R group. In patients with a RAS mutation, mOS was 25.4 months in the VEGF L group and 19.4 months in the VEGF R group (*P*=0.167). Judicious treatment allocation in Taiwanese patients with mCRC can result in an mOS of 34.3 months using cetuximab plus chemotherapy for left-sided tumors. An mOS of 48.5 months can be achieved using cetuximab plus chemotherapy in the neoadjuvant setting in mCRC patients with left-sided tumors. This study expands our understanding of the role of target therapy in improving survival of mCRC patients based on real-world study results.

## 1 Introduction

Colorectal cancers (CRCs) are the third most common type of cancer diagnosed globally, comprising 11% of all cancer diagnoses (GLOBOCAN 2018) (1). In 2018 CRC was associated with approximately 881,000 deaths (2) which made it the second deadliest cancer worldwide. In Taiwan, CRC has been the most commonly diagnosed cancer for many years and ranked third among men and women in terms of mortality rate in 2018 (3, 4). The incidence of CRC in Taiwan is increasing and has been especially rapid in persons older than 50 years of age (5). Despite advancements in therapy, nearly half of CRC patients develop metastatic disease, which is a major cause of death and associated with a 5‐year survival rate (6). The management of mCRC is continuously evolving with advancements in therapies, including surgery and radiotherapy. Target therapy has greatly improved the treatment paradigm of CRC. Many factors like tumor location, genetic variance and therapy sequence can further help to stratify suitable patients for optimal treatment, thereby further enhancing clinical efficacy and outcome.

Only 10% of “real-world” patients are represented in most randomized clinical trials (7). The remaining 90% of patients are underrepresented in clinical trials, including those with significant comorbidities, those living in remote regions, and those belonging to advanced age groups. The aim of this study was to evaluate the efficacy of target therapy in combination with chemotherapy for mCRC in a real-world setting to provide a macro-view of the treatment response in a local population in Taiwan. It was supposed that the real-world outcome would be like the clinical trials and determine what if the outcome is not as aspected. With this large observational study compared with the clinical trial data, we could know the difference between the trial and the reality and adjust the treatment police by the study.

## 2 Materials and methods

### 2.1 Study Design and Patient Population

This was a real-world, prospectively collected retrospective review study of 1583 patients with mCRC collected during routine management at the Taipei Veterans General Hospital (TPEVGH) in Taipei, Taiwan between 2005 and 2017. Patients were included if they: (a) had confirmed cases of mCRC through both imaging and pathological examination; (b) were candidates for anti‐EGFR therapy or anti‐VEGF therapy in combination with cytotoxic chemotherapy (FOLFOX or FOLFIRI); and (c) had measurable lesions (visible) before starting therapy. Both RAS wild type and RAS mut patients were included.

Patients were divided into four groups based on the location of the primary tumour and the use of the first-line target therapy. Patients treated with anti-EGFR who had right-sided tumor (EGFR R; tumors located between the caecum and the transverse colon), patients treated with anti-EGFR in the first-line who had left-sided tumor (EGFR L; tumors within the splenic flexure, descending colon, sigmoid colon and rectum), patients treated with anti-VEGF who had right-sided tumor (VEGF R), and patients treated with anti-VEGF who had left-sided tumor (VEGF L). Patient follow up continued until the patient expired or lost contact. Study data was collected over a period of 12 years.

### 2.2 Clinical Outcome Assessment

Responses to therapy were evaluated using computed tomography (CT) based on Response Evaluation Criteria in Solid Tumors (RECIST) criteria (version 1.1) (5).Tumor markers with carcinoembryonic antigen (CEA) and carbohydrate antigen 19-9 (CA19-9) were also used to evaluate the treatment response and disease behaviour. Overall survival (OS) was calculated from the starting date of therapy to the time of death due to any cause (expressed as median). Progression-free survival (PFS) was calculated from the treatment start date until tumour progression or death. For patients who were alive at final analysis, data on survival were censored at the last contact.

### 2.3 Molecular Analysis

Molecular analysis of mCRC tumor samples was routinely performed by the pathology department at TPEVGH. DNA was extracted from tissue sections (10 μm)prepared from formalin-fixed paraffin-embedded tumor samples using the QIAamp DNA FFPE Tissue Kit (Qiagen). Molecular testings were conducted by using *KRAS/BRAF* Mutation Analysis Kit for Real-Time PCR (*KRAS* exons 2, 3, 4 and *BRAF*600; EntroGen) and the *NRAS* Mutation Analysis Kit (exons 2, 3, and 4; EntroGen) which were approved for *in vitro* diagnosis. These polymerase chain reaction (PCR)–based assays use allele-specific probes to identify *KRAS* (18 mutations), *NRAS* (11 mutations), and *BRAFV*600 mutations, with the detection limit of less than 1%.

### 2.4 Statistical Analysis

The baseline characteristics of the included patients and the response data are presented as n (%). OS was expressed as a median (months) and estimated using the Kaplan‐Meier method and log‐rank test. All of the statistics were performed using the SPSS statistical 24.0 (SPSS Inc., Chicago, IL). Statistical significance was recognized at *P*<0.05.

## 3 Results

### 3.1 Patients

A total of 1583 mCRC patients from Taipei Veterans General Hospital in Taiwan were included in the study, 792 patients received chemotherapy plus target therapy and 791 patients received chemotherapy alone. Among those who received target therapy, 180 patients received chemotherapy with anti-EGFR therapy, and 612 patients received chemotherapy with anti-VEGF therapy.

A total of 635 (40.1%) of the patients were female and 948 (59.9%) were male, with a mean age of 63.3 years (range: 22-96 years). Patients had an ECOG performance status of 0 (n=1029 [65%]), 1 (n=298 [19%]), and 2 (n=87 [6%]). Tumors were localized to the left-side of the colon in 1124 (71%) patients, 477 (30%) to the right-side of the colon, and 106 (7%) had tumors localized to rectum. A total of 1068 (67%) patients had liver metastases, 600 (38%) had lung metastases, and 207 (13%) had peritoneum metastases. Of the 1583 mCRC patients, 171 (10.8%) patients refused further treatment, 1412 (89%) patients received first-line chemotherapy, 1051 (66%) patients received second-line chemotherapy, 697 (44%) patients received 3^rd^ line chemotherapy, and 401 (25%) patients received 4^th^ line chemotherapy (**Table 1**). The mean duration of follow up was 25.32 months.

**Table 1:** Baseline characteristics of the patients included in the study.

	N = 1583	
Gender		
Male	948	(59.9%)
Female	635	(40.1%)
Age (y, range)	63.3	(22-96)
ECOG		
0	1029	(65%)
1	298	(19%)
2	87	(6%)
Location		
Left colon	1124	(71%)
Right colon	477	(30%)
Rectum	106	(7%)
Metastasis		
Liver	1068	(67%)
Lung	600	(38%)
Peritoneum	207	(13%)
Chemotherapy		
1^st^	1412	(89%)
2^nd^	1051	(66%)
3^rd^	697	(44%)
4^th^	401	(25%)
Follow up (median, months)	25.32	

Patients were divided into EGFR R, RGFR L, VEGF R and VEGF L groups based on the location of the primary tumour and the use of the first-line target therapy. There were 47 patients in EGFR R group, 133 patients in EGFR L group, 196 in VEGF R group and 416 in VEGF L group. The characteristics of age, gender, ECOG, metastatic sites and further metastasectomy and Ras mutation are shown in **Table 2**.

**Table 2:** The patients’ characteristics in the groups by different tumor location and target therapy.

n	EGFR R		EGFR L		VEGF R		VEGF L	
	47		133		196		416	
Gender								
Male	20	(42.6%)	84	(63.2%)	100	(51.0%)	257	(61.8%)
Female	27	(57.4%)	49	(36.8%)	96	(49.0%)	159	(38.2%)
Age (y)	65	59	66	62		
ECOG								
0	19	(40.4%)	90	(68.2%)	138	(70.8%)	308	(74.0%)
1	12	(25.5%)	19	(14.4%)	45	(23.1%)	81	(19.5%)
2	5	(10.6%)	7	(5.3%)	7	(3.6%)	15	(3.6%)
Metastasis								
Liver	32	(68.1%)	102	(76.7%)	131	(66.8%)	292	(70.2%)
Lung	17	(36.2%)	40	(30.1%)	56	(28.6%)	167	(40.1%)
Metastasectomy after neoadjuvent	16	(34.0%)	53	(39.8%)	42	(21.4%)	125	(30.0%)
Ras mutation	1	(2.1%)	0	0	83	(42.3%)	174	(41.8%)
Overall survival (median, m)	13.8	35.7	18.7	26.9

### 3.2 CRC Location and First-Line Therapy

**Figure 1:**
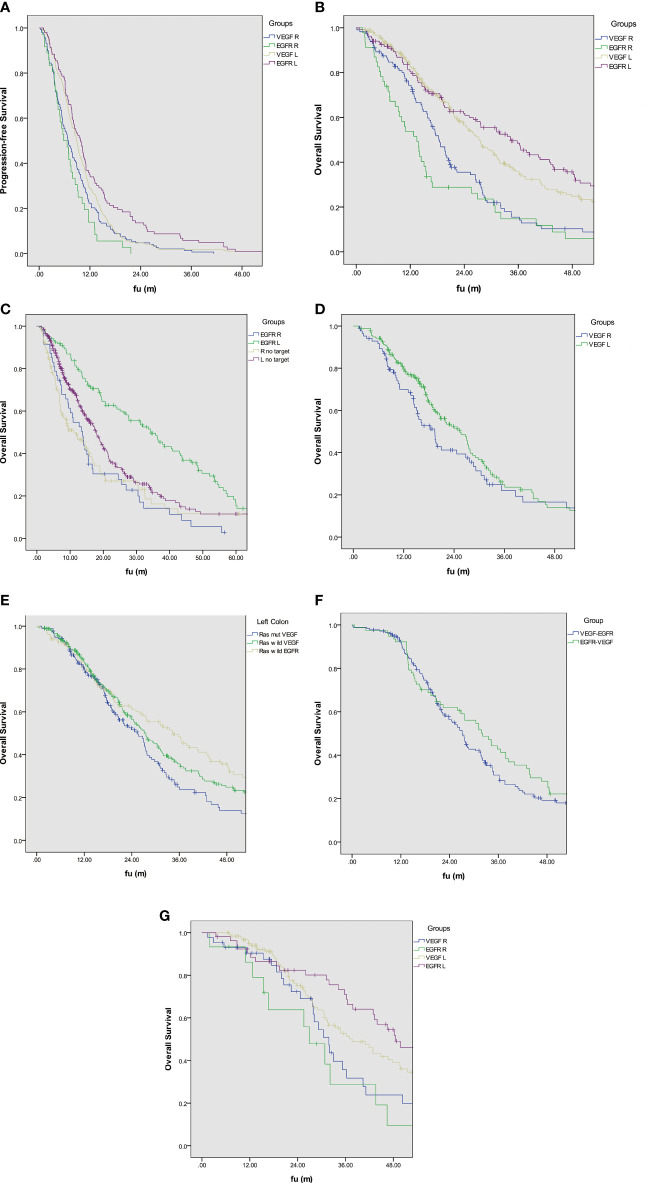
**(A)** Kaplan–Meier estimates of progression-free survival based on target therapy and sidedness in Ras wild type mCRC. **(B)** Kaplan–Meier estimates of overall survival based on target therapy and sidedness in Ras wild type mCRC. **(C)** Kaplan–Meier estimates of overall survival with anti-EGFR therapy based on sidedness in Ras wild type mCRC. **(D)** Kaplan–Meier estimates of overall survival with anti-VEGF therapy based on sidedness in Ras mutation mCRC. **(E)** Kaplan–Meier estimates of overall survival based on target therapy and Ras mutation status in left-sided mCRC **(F)** Kaplan–Meier estimates of overall survival based on the sequence of target therapy. **(G)** Kaplan–Meier estimates of overall survival based on neoadjuvant target therapy and sidedness before metastasectomy.

In RAS wild type patients, median PFS was 9.8 months in the EGFR L group, 8.9 months in the VEGF L group, 6.8 months in the VEGF R group, and 5.8 months in the EGFR R group (**Figure 1A**). OS was 34.3 months in the EGFR L group, 27.3 months in the VEGF L group, 18.4 months in the VEGF R group, and 13.5 months in EGFR R group (**Figure 1B**). PFS and OS were significantly different between the groups (*P*<0.001). These data suggest that primary tumor sidedness and target therapy selection significantly impact PFS and OS in RAS wild-type Taiwanese mCRC patients.

Comparing the anti-EGFR effect on the Ras wild type mCRC, the median OS showed better outcomes than those without target therapy who received chemotherapy only (**Figure 1C**). The left-sided mCRC had median OS 34.3 months better than those without any target therapy with a median OS of 17.9 months. The right-sided mCRC had a median OS of 13.5 months, which is better than those without any target therapy with a median OS of 11.1 months. Ths survival between these four groups had statistically significantly (*P*<0.001).

In patients with a RAS mutation (including K-Ras and N-Ras), median OS was 25.4 months in the VEGF L group and 19.4 months in the VEGF R group (*P*=0.144) (**Figure 1D**).

When comparing patients with left-sided CRC, median OS was 34.3 months in the RAS wild-type EGFR group, 27.3 months in the RAS wild-type VEGF group, and 25.4 months in the RAS-mut VEGF group. The difference in OS was not statistically significant (*P*=0.134) between groups, despite an OS nearly 9 months greater in the RAS wild-type EGFR group compared to the RAS-mut VEGF group (**Figure 1E**).

### 3.3 Target Therapy Sequence of the First-Line Therapy

Patients with left-sided RAS wild-type mCRC who received first-line anti-EGFR followed by anti-VEGF were grouped as EGFR-VEGF and vice versa. The OS was 32.1 months in the EGFR-VEGF group, slightly greater than the 27.1 months in the VEGF-EGFR group (*P*=0.43) (**Figure 1F**).

### 3.4 Target Therapy Before the Metastasectomy

A total of 315 (19.9%) patients received surgical treatment for the primary lesion and the associated metastasis. OS in these patients was 46.3 months compared with 19.2 months in those patients who did not receive surgical treatment for metastatic lesions (*P*<0.001). A total of 236 patients received target therapy before the metastasectomy. The median OS was 48.5 months in the EGFR L group, 37.6 months in the VEGF L group, 31.8 months in VEGF R group, and 26.9 months in the EGFR R group (*P*=0.021) (**Figure 1G**).

## 4 Discussion

The evolving advancement in the management of mCRC, such as the use of target therapies have increased OS by 24-30 months in patients with mCRC (8, 9). Cetuximab, Panitumumab, and Bevacizumab are the current first-line target therapies for mCRC currently reimbursed when used in combination with different chemotherapies by Taiwan National Health Insurance. In this study we aimed to evaluate the efficacy of target therapy in combination with cytotoxic chemotherapy for mCRC in a real-world setting.

### 4.1 Impact of Target Therapy and Sidedness

It has been increasingly recognized that right- and left-sided CRC differ in their clinical characteristics, anatomic structure, embryological origin, and the genetic mutation profile (10, 11). Left versus right sidedness also has a significant impact on the response to target therapy and patient survival (12). In this study, the difference between the PFS and OS were significant between groups (*P*<0.001), suggesting that sidedness and first-line target therapy significantly impact PFS and OS in the examined population in Taiwan. Therefore, these factors are important to consider when making treatment decisions for patients with mCRC.

In 2013, a Taiwanese study (13) showed that Taiwanese patients with mCRC with a KRAS wild-type respond better to a cetuximab plus chemotherapy regimen. Considering the target therapy in mCRC, our study further demonstrated that in the wild-type RAS patients, those with left-sided tumors derived the maximum benefit compared to patients with right-sided tumors (34.3 months versus 13.5 months). This is also in line with a previous meta-analysis of 13 first-line mCRC clinical trials and one prospective pharmacogenetic study that demonstrated patient’s with left-sided tumors had significantly better OS compared to patients with right-sided tumors (HR: 0.69 vs. 0.96). Moreover, significantly greater survival was observed in patients with left-sided primary tumors treated with anti-EGFR compared with anti-VEGF when added to standard chemotherapy (HR: 0.71; p=0.0003). In contrast, anti-VEGF based treatments were associated with better survival in patients with right-sided tumors (HR:1.3; P=0.081) (14). However, in our study population, the anti-EGFR therapy to right-sided mCRC still showed some benefit to those without any target therapy, but the benefit is not much as anti-VEGF therapy in right-sided mCRC.

Based on the collective findings from these studies, NCCN currently recommends cetuximab and panitumumab for the first‐line treatment of left‐sided tumors, when used in combination with cytotoxic agents in *RAS*–wild type mCRC. Anti-VEGF therapy may be preferred for right‐sided tumors in this setting due to a reported improved response (14).

Our results suggest improved outcomes for patients with RAS wild type left-sided CRC who received first-line anti-EGFR therapy, although no statistical significance was shown, which may have been impacted by the difference in the sample size of each arm (the sample size of RAS wild type VEGF group was larger than the RAS wild type EGFR group).

### 4.2 Target Therapy Sequence

Determining treatment sequences for patients with mCRC, especially target therapy, remains a therapeutic dilemma for oncologists. In terms of cytotoxic therapy both FOLFOX and FOLFIRI are considered equivalent; however, the optimal sequence of target therapy remains unknown. Furthermore, there are no established guidelines for the target therapy sequence of cytotoxic or target agents in mCRC. In the current study, OS was 32.1 months in the group receiving cetuximab followed by bevacizumab and 27.1 months in the group receiving bevacizumab followed by cetuximab. Taken together, a 5-month non-significant difference (*P*=0.43).

#### 4.2.1 First Line

The FIRE‐3 study (n= 592) examined cetuximab as first-line therapy in patients with mCRC carrying KRAS exon 2 wild-type. Statistical differences were not observed in ORR (62.0% vs. 58.0%; *P*=0.18) or median PFS (PFS; 10.0 vs. 10.3 months; *P*=0.55); however, median OS favored the cetuximab arm over bevacizumab (28.7 vs. 25.0 months; *P*=0.017) *(* 15, 16). In the FIRE-3 *post-hoc* analysis reported in 2016 showed that in mCRC patients with final RAS wild-type (KRAS/NRAS exon 2-4), the combination of cetuximab with chemotherapy as first‐line treatment resulted in higher OS than the combination of bevacizumab with chemotherapy (33.1 vs. 25.6 months; *P*=0.011) *(* 17). In addition, a secondary analysis with subsequent lines of therapy showed that patients who started cetuximab versus bevacizumab during first‐line therapy demonstrated longer PFS (6.5 vs. 4.7 months; *P*<0.001) and OS (16.3 vs. 13.2 months; *P*=0.0021) from the start of second‐line therapy (15, 16).

#### 4.2.2 Optimal Treatment Sequence

The optimal treatment sequence of anti-EGFR and anti-VEGF and the impact of first-line target therapy on second-line therapy have been examined in previous studies. In a retrospective analysis, Hsu HC et al. (15)reported increased OS patients treated with cetuximab followed by bevacizumab compared to patients treated with bevacizumab followed by cetuximab (median OS: 30.4 vs 25.7 months; *P*=0.008). Second‐ and third‐line OS was also higher in patients treated with cetuximab followed by bevacizumab compared to patients treated with bevacizumab followed by cetuximab (second‐line OS: 20.6 vs 14.8 months; *P*=0.004; third‐line OS: 12.5 vs 9.9 months; *P*=0.005). A retrospective analysis by Liu et al. (18)reported similar results in 101 left-sided RAS wild-type mCRC patients. Briefly, 50 cases received bevacizumab plus chemotherapy in both first- and second-line therapies (Group A) and 51 cases received first-line cetuximab plus chemotherapy followed by second-line bevacizumab-containing regimens (Group B). PFS1 was comparable (10.0 vs 10.4 months; *P*=0.402), while PFS2 (4.6 vs 7.9 months; *P*=0.002), OS1 (26.8 vs 40.0 months; *P*=0.011), and OS2 (15.2 vs 22.3 months; *P*=0.006) were reduced in Group A compared with Group B. A recent *in-vitro* study (19) compared the sequential administration of either cetuximab followed by bevacizumab (CET-BEV) or bevacizumab followed by cetuximab (BEV-CET) in a LIM1215 (KRAS wild type) and SW948 (KRAS mutant) xenograft mouse model. These results suggest that bevacizumab may act through the modulation of the tumor microenvironment by inducing hypoxia and cetuximab administration prior to bevacizumzb could trigger protective effects. In both LIM1215 and SW948 xenograft models, the survival benefit with cetuximab and bevacizumab monotherapy was observed; however, only the sequence CET-BEV was associated with an additional benefit.

### 4.3 Overall Survival in Patients Undergoing Metastasectomy

Target therapies have been evaluated in previous studies as potential adjuncts for conversion chemotherapy for mCRC. Prognosis depends on the extent of metastatic spread as the probability of undergoing potentially curative surgical resection of the metastatic lesions directly impacts survival. Resected patients with liver mCRC have demonstrated a median survival of 3.6 years (20).

Per NCCN and ESMO guidelines conversion management should be considered for unresectable mCRC in order to improve prognosis. Dependent upon which presurgical therapy was administered in the current study prior to metastasectomy, the difference in OS was significant (*P*=0.021) in terms of sidedness and target therapy. Therefore, we were able to confirm favorable long-term survival for patients with initial sub-optimal or unresectable colorectal liver metastases who responded to cetuximab-based conversion therapy and underwent secondary resection.

## 5 Conclusions

Judicious treatment allocation in patients with mCRC can be associated with an OS of up to 34.3 months when first-line cetuximab plus chemotherapy is used to treat Taiwanese patients with left-sided mCRC. Furthermore, an OS up to 48.5 months can be achieved when cetuximab plus chemotherapy is administered in the neoadjuvant setting for left-sided CRC in patients who received primary tumor resection and metastasectomy. Since the eligibility criteria for the current study were not restricted, it is reasonable to suggest these data represent the survival patterns of Taiwanese patients with mCRC in clinical practice.

## Data Availability Statement

The original contributions presented in the study are included in the article/supplementary material. Further inquiries can be directed to the corresponding author.

## Ethics Statement

This study was approved by the Institutional Review Board of Taipei Veterans General Hospital (TPEVGH IRB No.: 2020-05-013AC). The patients/participants provided their written informed consent to participate in this study.

## Author Contributions

Conception and design: S-CH, C-CL, H-WT, H-HL, S-CC, Y-TL, H-SW, S-HY, W-SC, and J-KJ. Acquisition of data: S-CH, C-CL, H-WT, H-HL, S-CC, Y-TL, H-SW, S-HY, W-SC, and J-KJ. Analysis and interpretation of data S-CH, C-CL, and H-WT, H-HL, S-CC, Y-TL, H-SW, S-HY, W-SC, and J-KJ. Drafting of the manuscript: S-CH and J-KJ. All authors contributed to the article and approved the submitted version.

## Conflict of Interest

The authors declare that the research was conducted in the absence of any commercial or financial relationships that could be construed as a potential conflict of interest.

## Publisher’s Note

All claims expressed in this article are solely those of the authors and do not necessarily represent those of their affiliated organizations, or those of the publisher, the editors and the reviewers. Any product that may be evaluated in this article, or claim that may be made by its manufacturer, is not guaranteed or endorsed by the publisher.

## Abbreviations

BEV, bevacizumab; CA19-9, carbohydrate antigen 19-9; CEA, carcinoembryonic antigen; CET, cetuximab; CI, confidence interval; CRC, colorectal cancer; CT, computed tomography; ECOG, Eastern Cooperative Oncology Group; EGFR, epidermal growth factor receptor; ESMO, European Society for Medical Oncology; HR, hazard ratio; mCRC, metastatic colorectal cancer; NCCN, National Comprehensive Cancer Network; ORR, objective response rate; OS, overall survival; RECIST, Response Evaluation Criteria in Solid Tumors; PFD, progression-free survival; VEGF, vascular endothelial growth factor.
